# Hospital Finance

**Published:** 1922-10

**Authors:** 


### HOSPITAL FINANCE.

### THE MAUDSLEY HOSPITAL SCHEME.

AFTER many unavoidable delays, for which the war was largely responsible, the London County Council has approved the report presented on the Maudsley Mental Hospital, Denmark Hill. Dr. Henry Maudsley, who died in 1918, had ten years earlier presented the L.C.C. with £30,000 for a hospital within five miles of Charing Cross, designed for early, non-certified cases and for mental research. To carry out this scheme by the Council, the legal anomaly preventing it from receiving voluntary boarders had to be removed by Parliament. The Council was also empowered to defray the whole cost of their treatment. At first it was thought desirable that certified cases should not be received, that the distinction might be emphasised. The remaining objects of the foundation were, as stated, medical research and the provision of an educational centre for medical students. But these aims have been felt to be hindered if admission were confined to voluntary boarders. Therefore the Council has endorsed its committee's recommendation that power be sought from the Government to receive, without a certificate, certifiable cases, with proper safeguards. Admission will be limited to residents in London, drawn from the out-patients' department, poor law institutions and private persons. The out-patients' department is expected to become self-supporting. To secure individual treatment, which is regarded as essential, a large medical staff is required. In fact, for a full complement of patients and staff, the cost of maintenance is estimated at £37,500 a year.

The hospital provides for 144 patients, or, including 13 rooms for paying patients, 157 beds. Thus there is ample accommodation for the free treatment of the poor and for those who, or whose friends, can only contribute modestly. It will be remembered that the site finally chosen was at Denmark Hill, which was bought for £10,000. When the buildings were nearly complete, in 1915, the War Office took them over in connection with the adjacent Fourth General Military Hospital. The main buildings were handed over in 1916. The hospital was demobilised in August, 1919, when the Ministry of Pensions acquired it, and held possession till August, 1920. Only lately have the protracted negotiations over the return of the buildings been completed. The pathological laboratory, when finished, was occupied by the director, who transferred his staff and equipment thereto from Claybury Mental Hospital. Dr. Maudsley left £10,000 to the Council to endow research at his foundation.

### A FAR-SIGHTED FINANCIER.

WHEN Mr. David Ainslie, of East Lothian, died in 1900, he left a large sum with the instruction that it should be allowed to accumulate for fifteen years. The grand total so obtained was to be spent upon the Astley-Ainslie Institution, endowed and built for the convalescent patients of the Royal Infirmary, Edinburgh. The war delayed matters, but now the trustees have purchased four houses and a former women's golf course. The property consists of 25 acres near Grange Loan, Edinburgh. Though the capital is said to amount to half a million, the trustees are wisely limiting their expenditure, in order that the institution shall be self-supporting. One house will accommodate 60, solely women, patients, and should be ready early next year. Two new wings are now in building. When the other houses are occupied, 350 convalescents will be accommodated. The cost of the land and houses is said to have been £70,000. There can be no question that these beds will relieve the demands on the Royal Infirmary and reduce the waiting list. In August the number waiting admission was 1,269, the highest ever recorded in the hospital's history. That the new beds should be self-supporting from the start is an enormous advantage, and the delay of fifteen years by which such a big institution has been financed proves Mr. Ainslie to have been a far-sighted hospital benefactor.

### A REVOKED BEQUEST.

WHEN a bequest to a hospital is revoked in a codicil it is very rarely because the testator has come to disapprove of the institution. The usual reason is to show public disapproval of the pressure of taxation upon the class to which the testator happens to belong. But the Corbett Hospital, Stourbridge, has lately lost a legacy of £100 for a curious reason. The late Mr. William Blow Collis, J.P., of Swinford House, Stourbridge, a consulting engineer, left a codicil revoking a legacy of £100 to the Corbett Hospital, "to mark my disapprobation of the action of the board of management of the said hospital in building a ward for the treatment of venereal disease in front of the men's ward of the hospital, in the belief that such action of the board of management is contrary to the expressed desire of the founder of the hospital, Mr. John Corbett." Perhaps Mr. J. Donaldson Harward, the hospital's secretary, can throw some light on this curious codicil.

### WHIST AND CHARITY.

IN order to raise the additional £3,000 a year needed by the Royal Sussex County Hospital, Brighton, Mr. J. Jonas has lately been trying to persuade that hospital to adopt the St. Dunstan's National Whist Championship Scheme. Though the hospital did not feel able to finance the scheme, it is worth describing, for other hospitals may care to consider it. For this reason Mr. Jonas's detailed plan for applying the scheme to Sussex may be briefly summarised. He estimated that 5 per cent. of the population of the county would enter for the championship (that is, 35,000 out of an aggregate of 750,000), and therefore participate in the first round; that 10 per cent. would qualify for the second or third, *viz.*, 3,500; that 10 per cent. would qualify for the final round, *viz.*, 350. The grand total would therefore be 38,850, or, " say, 40,000 at 2s. each." The gross receipts would therefore be £4,000, and with expenses estimated at £1,000 this would leave £3,000 for the hospital from what might become an annual event. The author, by the way, had secured some excellent prizes, including £100 from a trading house, and a week's motor-coach tour in England with hotel expenses included. He suggests that if a generous donor had offered, or would offer, to give the £1,000 necessary to start the scheme, the hospital would have accepted it. That would be the ideal solution, since the plan cannot guarantee its own success. But in these days, when the business side of charity is sedulously cultivated, it is worth considering, especially when one remembers how fertile St. Dunstan's has proved in similar ideas. The " Sussex Daily News," which gave the proposer space to elaborate his version, may yet record the name of someone prepared to make the attempt feasible.

The rent roll of the Bridewell Hospital amounted last year to £29,608.

Extensions to Beckenham Hospital, at a cost of £18,000, are to be made.

By visiting the public houses in his parish, the Rev. Mark Johnson, vicar of Holy Trinity, Haverstock Hill, has collected £14 9s. for the Hospital Sunday Fund.

The shocks received from air-raids during the war having endangered the roof, balcony and coping of the Kilburn, Maida Vale and St. John's Wood Dispensary, £200 has had to be spent upon repairs.

The United Services Fund is opening at Heatherwood, Ascot, a hospital for surgical tuberculosis in the children of ex-Service men and women. Children will be admitted up to the age of 12. There is accommodation for 150 patients. The friends or relatives of patients will contribute according to their means.

After twenty-two years, Mr. F. Stanners has given £50 to the Coventry and Warwickshire Hospital in fulfilment of of the wish then expressed to show his gratitude. He had met with an accident on Thanksgiving Day, and after two months' treatment made a donation of £2 10s., which was all that his means then allowed.

Grants made by the League of Mercy to the hospitals within its area of collection, but outside the London area, amount this year to £5,170. The total grants awarded to extra-metropolitan hospitals amount to £49,066, including £170 to Indian hospitals. £324,000 has been paid over to King Edward's Hospital Fund for distribution to the hospitals within the London area.

A flag day to be known as " Our Day and the Prince's Day " is now being organised on behalf of the Joint Council of the Order of St. John and the British Red Cross Society and the Combined Hospitals of London, to take place on Friday, October 6. The area to be covered will be the County and the City of London—bordered on the north by Hampstead, Islington, Stoke Newington and Hackney ; on the south by Wandsworth, Lambeth, Camberwell and Lewisham ; on the east by Woolwich ; and on the west by Hammersmith.

Devonshire House has been offered to the Committee of the Hospitals of London Combined Appeal, for exhibition purposes, from mid-October to the end of December. Acceptance depends upon exemption from rates by the L.C.C. and the Westminster City Council, and it is estimated that if this is allowed the use of the house will ensure net proceeds of at least £10,000 towards the £190,000 which remains to be raised by the end of 1922. It is understood that demolition of Devonshire House will be begun early next year, as part of an improvement scheme which includes the widening of Berkeley Street.

